# Association between *Helicobacter pylori* infection, serum thyroid-stimulating hormone, and thyroxine in the National Health and Nutrition Examination Survey 1999–2000

**DOI:** 10.3389/fendo.2025.1482073

**Published:** 2025-02-03

**Authors:** Ting Lu, Shunshun Lu, Jieqiong Lin, Xiaona Shao, Dahua Chen, Jianwei Shen

**Affiliations:** ^1^ Department of Gastroenterology, Ningbo Medical Center Lihuili Hospital, Ningbo, ZheJiang, China; ^2^ Department of Hospital-Acquired Infection Control, Ningbo Medical Center Lihuili Hospital, Ningbo, ZheJiang, China

**Keywords:** *Helicobacter pylori* infection, thyroid stimulating hormone, thyroxine, NHANES, CDC

## Abstract

**Background:**

*Helicobacter pylori* has been increasingly implicated in extra-gastric diseases. Current evidence regarding the association between serum thyroid-stimulating hormone (TSH), thyroxine (T4), and *H. pylori* infection remains inconclusive. Consequently, this study aimed to explore the correlation between TSH and T4 levels and *H. pylori* infection in a US-based population sample.

**Methods:**

Data from the US National Health and Nutrition Examination Survey (NHANES), comprising 971 participants aged 30–85 years from 1999 to 2000, were analyzed. Binary logistic regression was employed to analyze the correlation between *H. pylori* and TSH and T4 levels. The impact of TSH and T4 on *H. pylori* infection was further assessed using restricted cubic spline (RCS) analysis. In addition, subgroup analyses stratified by sex and age were conducted.

**Results:**

Subjects with *H. pylori* seropositivity demonstrated lower serum TSH levels and higher serum T4 levels compared to those with *H. pylori* seronegativity. A significant positive correlation was identified between *H. pylori* seropositivity and T4 levels with increasing quartiles of hormonal levels in both univariate regression models (Q4 vs. Q1: OR = 1.483; 95% CI, 1.033–2.129) and multivariate regression models (Q4 vs. Q1: OR = 1.004; 95% CI, 0.981–1.026). Conversely, a negative correlation was observed between *H. pylori* seropositivity and TSH levels with increasing quartiles of hormonal levels in univariate regression models (Q4 vs. Q1: OR = 0.579; 95% CI, 0.403–0.831) and in multivariate regression models (Q4 vs. Q1: OR = 0.580; 95% CI, 0.389–0.866). In stratified analyses, the adjusted association of serum T4 levels with *H. pylori* seropositivity was statistically significant among men (T4: Q4 vs. Q1: OR = 2.253; 95% CI, 1.311–3.873), age over 68 years in TSH levels (Q4 vs. Q1: OR = 0.434; 95% CI, 0.206–0.911), and age 41–54 years in T4 levels (Q4 vs. Q1: OR = 4.965; 95% CI, 2.071–11.903). RCS analysis revealed a non-linear relationship between TSH levels and *H. pylori* infection. Notably, when TSH < 0.98 IU/ml, the likelihood of *H. pylori* infection significantly increased.

**Conclusions:**

Lower TSH and higher T4 levels were associated with *H. pylori* infection, particularly among men and elderly individuals.

## 1 Introduction

*Helicobacter pylori* is a bacterium that preferentially colonizes in the gastric epithelium, often causing a spectrum of digestive disease, including peptic ulcer, chronic gastritis, and even gastric cancer. It has been reported that approximately half of the global population is infected with *H. pylori* (1), with a prevalence rate of approximately 35.6% in the US (2). Furthermore, numerous extra-gastric diseases have been proven to be associated with *H. pylori* infection, such as dermatosis, thyroid diseases, and metabolic, cardiovascular, and neurological diseases (3–5).

Thyroxine (T4) is a thyroid hormone (TH) synthesized and secreted by the thyroid gland (6). The regulation of THs is governed by the complex hypothalamic–pituitary–thyroid (HPT) axis. Thyroid-stimulating hormone (TSH), produced by the anterior pituitary gland, promotes the synthesis and release of THs, primarily through negative feedback mechanisms within the HPT axis (7). Evidence indicates that *H. pylori* infection plays a pivotal role in thyroid disease (8), particularly autoimmune thyroid diseases (ATDs), owing to its ability to mimic the antigenic profile present on thyroid cell membranes (9). Vincenzo Bassi et al. discovered a heightened prevalence of *H. pylori* exclusively among patients with hyperthyroid Graves’ disease (GD), in contrast to those with Hashimoto thyroiditis (HT) (10). Additionally, De Luis et al. reported markedly elevated levels of anti-*H. pylori* immunoglobulin G antibodies in patients with subclinical hyperthyroidism, compared to the control cohort (11). Furthermore, a recent prospective study highlighted a substantial association between *H. pylori* infection and the likelihood of subclinical hyperthyroidism in Chinese women, independent of dietary factors (12). Both TSH and T4 are considered sensitive biomarkers for evaluating thyroid function, reflecting conditions of either hypothyroidism or hyperthyroidism (13). At present, the relationship between *H. pylori* infection and plasma levels of TSH and T4 in the general population remains insufficiently investigated and contentious (14–16).

Since the T4 data were incorporated in October 2023, it is now particularly intriguing to explore the relationship between TSH, T4 levels, and *H. pylori* infection based on data from the 1999–2000 National Health and Nutrition Examination Survey (NHANES).

## 2 Materials and methods

### 2.1 Study design and sample

NHANES is a publicly accessible database managed by the Centers for Disease Control and Prevention (CDC), providing extensive data regarding the health and nutritional status of the non-institutionalized U.S population. The survey encompasses information derived from questionnaires, demographic profiles, laboratory tests, and physical examinations (17, 18). The data analyzed in this study were form the 1999–2000 NHANES cycles, encompassing participants who had both *H. pylori* infection and measurements of plasma TSH and T4 levels, with T4 data being added to the database in October 2023.

**Figure 1:**
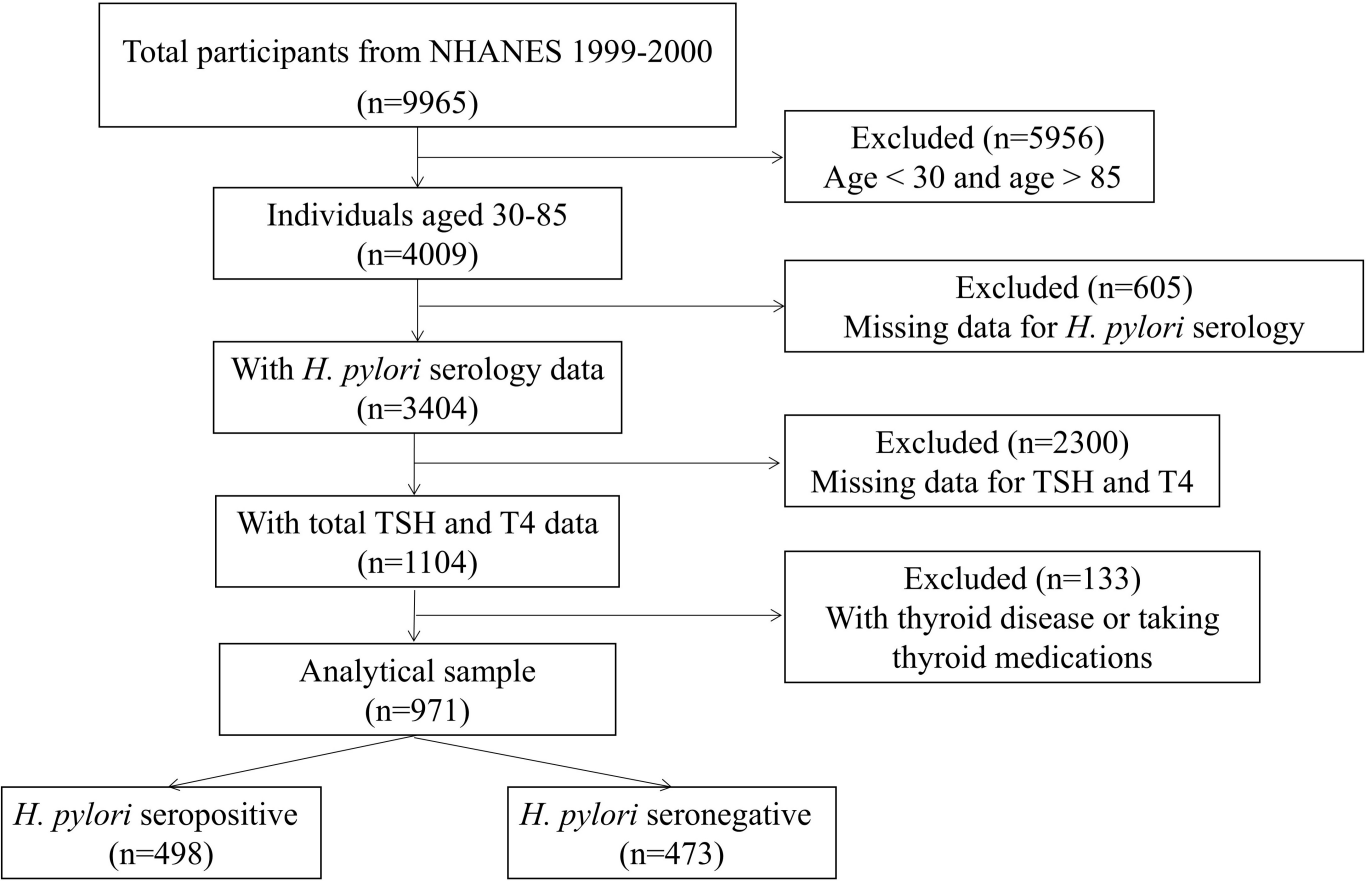
The flowchart of sample selection.

The exclusion criteria were as follows: (1) individuals aged <30 or >85 years; (2) missing data for *H. pylori* serology, TSH, or T4 levels; and (3) individuals with thyroid disease or taking thyroid medications. Participants possessing relevant laboratory data and demographic variables of interest were incorporated into this investigation, culminating in a total sample size of 971 individuals aged between 30 and 85 years old. The schematic representation of the participant selection process is illustrated in **Figure 1**.

### 2.2 *Helicobacter pylori* status

In accordance with the NHANES protocol (19), *H. pylori*-specific immunoglobulin G (IgG) levels were quantified utilizing the *H. pylori* IgG enzyme-linked immunosorbent assay (ELISA) developed by Wampole Laboratories (Granbury, N) (20). Standard ELISA cutoff values were applied to classify participants as seropositive [optical density (OD) value ≥1.1] or seronegative (OD value < 0.9) for *H. pylori*. Indeterminate results (OD values between 0.9 and 1.1) were excluded to avoid potential biases in the statistical analysis of this investigation (21).

### 2.3 Thyroid-stimulating hormone and thyroxine

The dependent variable analyzed in this study was *H. pylori* seropositivity, while the primary independent variables of interest were plasma levels of TSH and T4. Both serum TSH and T4 were sourced from the NHANES laboratory dataset, designated as LAB18T4, which was updated in October 2023. Thyroid medications have been identified in the prescription drug medication document.

### 2.4 Covariates

The covariates examined in this research encompass age, gender, race, education level, body mass index (BMI), smoking behavior, alcohol behavior, and homocysteine levels. These variables were selected based on existing evidence linking them to both *H. pylori* serostatus and thyroid function (18, 21, 22). Among the covariates, age, TSH, T4, and homocysteine were categorized as continuous variables, while sex, race, education level, BMI, smoking behavior, and alcohol behavior were classified as categorical variables.

### 2.5 Statistical analyses

For continuous variables, independent *t*-test or Mann–Whitney test was employed to analyze the differences between groups. Depending on the normality of distribution, continuous variables were presented as mean ± SD; otherwise, they were presented as Median. Categorical variables were assessed using the chi-square test and reported as counts and percentages. Levels of TSH and T4 were categorized into quartiles (Q1 to Q4). Multiple regression analysis was conducted to identify the factors influencing TSH and T4 levels. Furthermore, the relationship between TSH, T4, and *H. pylori* was examined through restricted cubic spline (RCS) analysis, with knots positioned at the 5th, 35th, 65th, and 95th percentiles. Statistical analyses were carried out using SPSS (version 26.0) and R software (version 4.1.3), with a significance threshold set at *p* < 0.05, where both the *p* for overall and the *p* for non linear relationship in RCS were less than 0.05.

## 3 Results

### 3.1 Characteristics of included subjects

A total of 971 participants were included in this study, with 498 classified as *H. pylori* IgG seropositive and 473 as *H. pylori* IgG seronegative. Notable differences were detected between the two groups (*p* < 0.05) in terms of age, race, educational level, and serum TSH and T4 levels. The baseline characteristics of the study subjects are detailed in **Table 1**.

**Table 1:** Baseline characteristics of the study subjects.

	*H. pylori* seropositive (*n* = 498)	*H. pylori* seronegative (*n* = 473)	*p*-value
Age (years)	56.0 (42.0–68.0)	52.0 (39.0–67.5)	**0.037**
Sex			0.168
Male	260 (52.2%)	226 (47.8%)	
Female	238 (47.8%)	247 (52.2%)	
Race			**<0.001**
Mexican American	197 (39.5%)	55 (11.6%)	
Other Hispanic	39 (7.8%)	9 (1.9%)	
Non-Hispanic white	132 (26.5%)	335 (70.8%)	
Non-Hispanic black	111 (22.2%)	65 (13.7%)	
Other races	19 (3.8%)	9 (1.9%)	
Educational level			**<0.001**
Less than high school	277 (55.6%)	108 (22.8%)	
High school	92 (18.4%)	120 (25.3%)	
More than high school	128 (5.7%)	244 (51.5%)	
Others	1 (0.2%)	1 (0.2%)	
BMI	27.78 (24.58–31.59)	27.27 (24.06–31.96)	0.317
Homocysteine (μmol/L)	8.14 (6.45–10.20)	7.69 (6.29–9.88)	0.055
Serum TSH (IU/mL)	1.48 (1.02–2.15)	1.64 (1.13–2.43)	**0.008**
Serum T4 (nmol/L)	97.80 (84.90–113.30)	92.70 (83.70–108.10)	**0.013**
Smoking behavior			0.21
Never	203 (40.8%)	194 (41.0%)	
Some days	113 (22.7%)	116 (24.5%)	
Every day	182 (36.5%)	163 (34.5%)	
Alcohol behavior			0.749
Yes	332 (66.7%)	322 (68.1%)	
No	166 (33.3%)	151 (31.9%)	

Other race/ethnicity includes all race/ethnicity other than Mexico-American, non-Hispanic white, and black. BMI, body mass index; TSH, thyroid-stimulating hormone; T4, thyroxine. Bold represents statistically significant.

### 3.2 Association between *H. pylori* seropositivity and TSH and T4

#### 3.2.1 Multiple regression model

The outcomes of different multivariate linear regression models are summarized in **Tables 2**, **3**: Model 1 is unadjusted, model 2 is adjusted for age and sex, and model 3 is further adjusted for race and educational level.

**Table 2:** Association of thyroid-stimulating hormone level with *Helicobacter pylori* seropositivity.

TSH	Model 1		Model 2		Model 3	
	OR (95% CI)	*p*-value	OR (95% CI)	*p*-value	OR (95% CI)	*p*-value
Q1	Ref		Ref		Ref	
Q2	0.731 (0.511–1.046)	0.087	0.715 (0.499–1.026)	0.068	0.701 (0.477–1.032)	0.072
Q3	0.780 (0.545–1.117)	0.175	0.758 (0.528–1.087)	0.132	0.775 (0.525–1.140)	0.195
Q4	0.579 (0.403–0.831)	0.003	0.544 (0.377–0.786)	0.001	0.580 (0.389–0.866)	0.008

Model 1: no covariates were adjusted; model 2: age and sex were adjusted; model 3: age, sex, race, and educational level were adjusted. OR, odds ratio. Q1: <1.09 mIU/L, Q2: 1.09–1.57 mIU/L, Q3: 1.57–2.30 mIU/L, Q4: >2.30 mIU/L.

**Table 3:** Association of thyroxine level with *Helicobacter pylori* seropositivity.

T4	Model 1		Model 2		Model 3	
	OR (95% CI)	*p*-value	OR (95% CI)	*p*-value	OR (95% CI)	*p*-value
Q1	Ref		Ref		Ref	
Q2	0.865 (0.601–1.245)	0.434	0.885 (0.614–1.278)	0.515	0.886 (0.597–1.316)	0.549
Q3	1.161 (0.810–1.666)	0.416	1.211 (0.840–1.747)	0.305	1.252 (0.842–1.862)	0.267
Q4	1.483 (1.033–2.129)	0.033	1.552 (1.076–2.238)	0.019	1.004 (0.981–1.026)	0.048

Model 1: no covariates were adjusted; model 2: age and sex were adjusted; model 3: age, sex, race, and educational level were adjusted. OR, odds ratio. Q1: <83.7 nmol/L, Q2: 83.7–95.2 nmol/L, Q3: 95.2–110.7 nmol/L, Q4: >110.7 nmol/L.

In the unadjusted model, a negative association was identified between *H. pylori* seropositivity and TSH levels across increasing quartiles of hormonal levels (Q4 vs. Q1: OR = 0.579; 95% CI, 0.403–0.831, *p* = 0.003). This negative relationship persisted after adjustments for confounding factors in model 2 (Q4 vs. Q1: OR = 0.544, 95% CI, 0.377–0.786; *p* = 0.001) and model 3 (Q4 vs. Q1: OR = 0.580, 95% CI, 0.389–0.866; *p* = 0.008). Conversely, a significant positive association was observed between *H. pylori* and T4 levels across increasing quartiles of hormonal levels (Q4 vs. Q1: OR = 1.483; 95% CI, 1.033–2.129, *p* = 0.033). This positive connection remained significant after adjustments for confounding factors in model 2 (Q4 vs. Q1: OR = 1.552; 95% CI, 1.076–2.238, *p* = 0.019) and was marginally significant in model 3 (Q4 vs. Q1: OR = 1.004; 95% CI, 0.981–1.026, *p* = 0.048), as presented in **Table 3**.

#### 3.2.2 Subgroup analyses

In the sex-stratified subgroup analyses, a positive correlation was discovered between the *H. pylori* seropositivity and T4 levels in men (Q4 vs. Q1 OR = 2.253; 95% CI, 1.311–3.873; *p* = 0.003). However, no significant relationship was observed between TSH levels and *H. pylori* seropositivity in men, as the positive correlation disappeared after adjusting for confounding variables in the multivariate regression model (Q4 vs. Q1 OR = 0.636; 95% CI, 0.340–1.188; *p* = 0.156). Among women, neither TSH nor T4 levels exhibited any association with *H. pylori* seropositivity. Detailed findings are provided in **Tables 4**, **5**.

**Table 4:** Association of thyroid-stimulating hormone level with *Helicobacter pylori* seropositivity based on subgroup of sex.

Subgroup	TSH	Model 1		Model 2		Model 3	
		OR (95% CI)	*p*-value	OR (95% CI)	*p*-value	OR (95% CI)	*p*-value
Male	Q1	Ref		Ref		Ref	
	Q2	0.621 (0.373–1.035)	0.068	0.614 (0.368–1.024)	0.062	0.611 (0.339–1.013)	0.102
	Q3	0.595 (0.355–0.997)	0.049	0.584 (0.348–0.980)	0.042	0.675 (0.370–1.233)	0.202
	Q4	0.494 (0.294–0.831)	0.008	0.465 (0.273–0.792)	0.005	0.636 (0.340–1.188)	0.156
Female	Q1	Ref		Ref		Ref	
	Q2	0.845 (0.508–1.406)	0.518	0.830 (0.498–1.385)	0.830	0.830 (0.449–1.436)	0.459
	Q3	1.012 (0.613–1.671)	0.963	0.974 (0.587–1.614)	0.918	1.248 (0.691–2.256)	0.463
	Q4	0.672 (0.405–1.116)	0.125	0.633 (0.379–1.058)	0.081	0.799 (0.440–1.452)	0.461

Model 1: no covariates were adjusted; model 2: age and sex were adjusted; model 3: age, sex, race, and educational level were adjusted. OR, odds ratio. Q1: <1.09 mIU/L, Q2: 1.09–1.57 mIU/L, Q3: 1.57–2.30 mIU/L, Q4: >2.30 mIU/L.

**Table 5:** Association of thyroxine level with *Helicobacter pylori* seropositivity based on subgroup of sex.

Subgroup	T4	Model 1		Model 2		Model 3	
		OR (95% CI)	*p*-value	OR (95% CI)	*p*-value	OR (95% CI)	*p*-value
Male	Q1	Ref		Ref		Ref	
	Q2	0.917 (0.570–1477)	0.722	0.937 (0.580–1.514)	0.791	1.169 (0.671–2.037)	0.582
	Q3	1.258 (0.773–2.047)	0.356	1.303 (0.795–2.135)	0.294	1.617 (0.913–2.864)	0.100
	Q4	2.184 (1.278–3.734)	0.004	2.253 (1.311–3.873)	0.003	2.061 (1.093–3.887)	0.025
Female	Q1	Ref		Ref		Ref	
	Q2	0.819 (0.459–1.461)	0.499	0.812 (0.454–1.454)	0.812	0.576 (0.298–1.112)	0.100
	Q3	1.097 (0.626–1.924)	0.746	1.142 (0.648–2.012)	0.645	0.938 (0.493–1.783)	0.844
	Q4	1.211 (0.702–2.088)	0.491	1.278 (0.737–2.216)	0.382	0.848 (0.447–1.610)	0.614

Model 1: no covariates were adjusted; model 2: age and sex were adjusted; model 3: age, sex, race, and educational level were adjusted. OR, odds ratio. Q1: <83.7 nmol/L, Q2: 83.7–95.2 nmol/L, Q3: 95.2–110.7 nmol/L, Q4: >110.7 nmol/L.

In the age-stratified subgroup analyses, a negative correlation was identified between *H. pylori* seropositivity and TSH levels among participants aged over 68 years (Q4 vs. Q1: OR = 0.434; 95% CI, 0.206–0.911; *p* = 0.027). Furthermore, no significant association was observed between TSH levels and *H. pylori* seropositivity in other age groups, as detailed in **Table 6**. Additionally, there was a positive association between *H. pylori* seropositivity and T4 levels among participants aged 41–54 years (Q4 vs. Q1: OR = 4.965; 95% CI, 2.071–11.903; *p* < 0.001). However, T4 levels showed no significant relationship with *H. pylori* seropositivity in other age groups, as outlined **Table 7**.

**Table 6:** Association of thyroid-stimulating hormone level with *Helicobacter pylori* seropositivity based on subgroup of age.

Subgroup	TSH	Model 1		Model 2		Model 3	
		OR (95% CI)	*p*-value	OR (95% CI)	*p*-value	OR (95% CI)	*p*-value
<41	Q1	Ref		Ref		Ref	
	Q2	0.729 (0.367–1.448)	0.366	0.730 (0.367–1.452)	0.730	0.409 (0.176–0.950)	0.038
	Q3	0.759 (0.397–1.591)	0.517	0.796 (0.397–1.593)	0.796	0.799 (0.339–1.880)	0.606
	Q4	0.622 (0.284–1.363)	0.235	0.621 (0.283–1.362)	0.235	0.658 (0.248–1.746)	0.401
41–54	Q1	Ref		Ref		Ref	
	Q2	0.806 (0.403–1.612)	0.541	0.804 (0.397–1.628)	0.544	1.551 (0.652–3.691)	0.321
	Q3	0.731 (0.358–1.493)	0.731	0.738 (0.357–1.527)	0.413	1.304 (0.529–3.212)	0.564
	Q4	0.505 (0.243–1.046)	0.066	0.570 (0.282–0.800)	0.139	1.198 (0.472–3.042)	0.704
54–68	Q1	Ref		Ref		Ref	
	Q2	1.031 (0.497–2.317)	0.953	1.041 (0.501–2.161)	0.914	0.911 (0.381–2.178)	0.835
	Q3	0.737 (0.356–1.526)	0.411	0.740 (0.357–1.533)	0.418	0.518 (0.210–1.279)	0.154
	Q4	0.650 (0.312–1.351)	0.248	0.811 (0.488–1.349)	0.420	0.712 (0.281–1.803)	0.474
>68	Q1	Ref		Ref		Ref	
	Q2	0.383 (0.171–0.861)	0.020	0.391 (0.174–0.880)	0.023	0.428 (0.178–1.030)	0.058
	Q3	0.722 (0.333–1.568)	0.411	0.719 (0.331–1.564)	0.406	1.153 (0.490–2.718)	0.744
	Q4	0.422 (0.202–0.884)	0.022	0.434 (0.206–0.911)	0.027	0.645 (0.283–1.470)	0.297

Model 1: no covariates were adjusted; model 2: age and sex were adjusted; model 3: age, sex, race, and educational level were adjusted. OR, odds ratio. Q1: <1.09 mIU/L, Q2: 1.09–1.57 mIU/L, Q3: 1.57–2.30 mIU/L, Q4: >2.30 mIU/L.

**Table 7:** Association of thyroxine level with *Helicobacter pylori* seropositivity based on subgroup of age.

Subgroup	T4	Model 1		Model 2		Model 3	
		OR (95% CI)	*p*-value	OR (95% CI)	*p*-value	OR (95% CI)	*p*-value
<41	Q1	Ref		Ref		Ref	
	Q2	0.436 (0.190–0.997)	0.049	0.434 (0.190–0.995)	0.049	0.288 (0.106–0.784)	0.015
	Q3	1.714 (0.792–3.712)	0.172	1.730 (0.797–3.758)	0.166	1.395 (0.539–3.612)	0.493
	Q4	1.062 (0.503–2.239)	0.875	0.921 (0.530–1.602)	0.835	0.749 (0.290–1.933)	0.550
41–54	Q1	Ref		Ref		Ref	
	Q2	2.500 (1.068–5.849)	0.035	2.974 (1.235–7.162)	0.015	2.711 (0.968–7.596)	0.058
	Q3	2.049 (0.911–4.610)	0.083	2.317 (1.006–5.336)	0.048	2.171 (0.782–6.030)	0.137
	Q4	4.083 (1.759–9.477)	0.001	4.965 (2.071–11.903)	<0.001	3.986 (1.346–11.798)	0.013
54–68	Q1	Ref		Ref		Ref	
	Q2	0.605 (0.292–1.256)	0.178	0.633 (0.302–1.324)	0.224	0.618 (0.252–1.513)	0.292
	Q3	0.646 (0.315–1.324)	0.233	0.683 (0.329–1.419)	0.307	0.853 (0.353–2.061)	0.724
	Q4	1.059 (0.515–2.178)	0.876	1.157 (0.546–2.454)	0.703	0.924 (0.360–2.373)	0.870
>68	Q1	Ref		Ref		Ref	
	Q2	1.026 (0.537–1.961)	0.937	0.975 (0.506–1.877)	0.939	1.134 (0.558–2.307)	0.728
	Q3	1.205 (0.590–2.459)	0.609	1.120 (0.542–2.316)	0.759	1.269 (0.584–2.759)	0.547
	Q4	1.564 (0.770–3.178)	0.216	1.446 (0.702–2.979)	0.318	1.306 (0.590–2.890)	0.510

Model 1: no covariates were adjusted; model 2: age and sex were adjusted; model 3: age, sex, race, and educational level were adjusted. OR, odds ratio. Q1: <83.7 nmol/L, Q2: 83.7–95.2 nmol/L, Q3: 95.2–110.7 nmol/L, Q4: >110.7 nmol/L.

#### 3.2.3 Non-linear relationship between TSH, T4, and *H. pylori* infection

**Figure 2:**
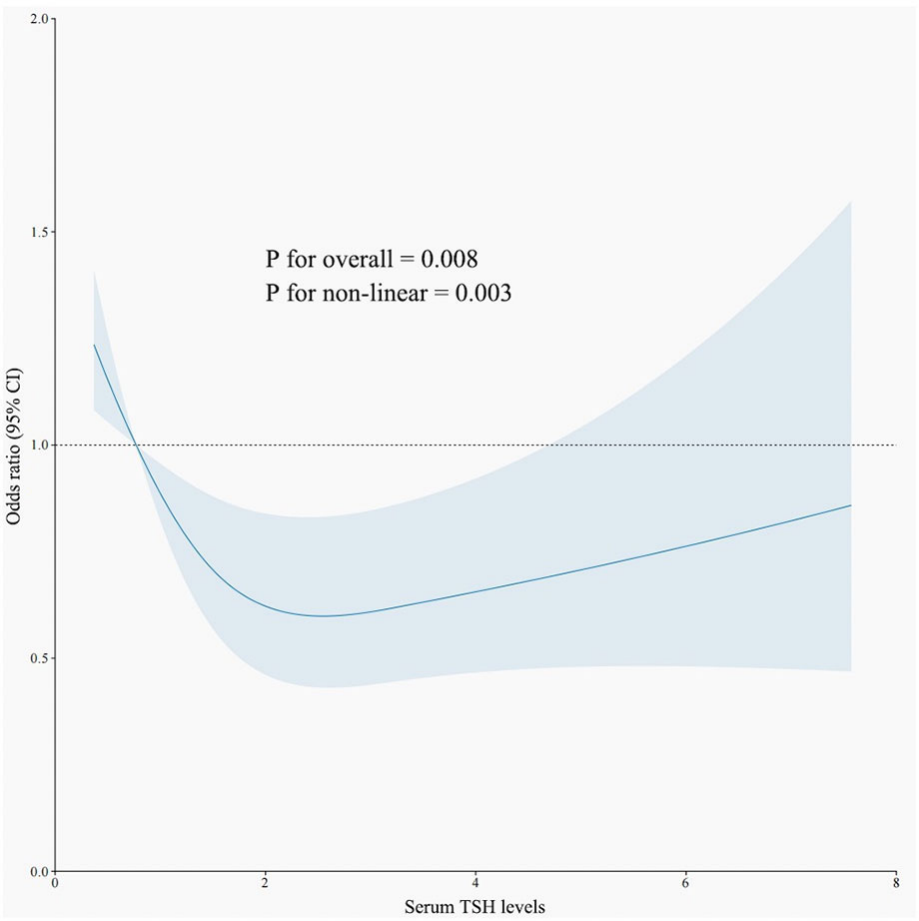
The restricted cubic spline curve for the relationship between serum TSH levels and *H. pylori* infection. The blue lines represent odds ratios, and blue areas represent 95% confidence intervals.

An RCS model was employed to assess the association between TSH levels and *H. pylori* infection. As illustrated in **Figure 2**, when TSH levels fall below 0.98 IU/mL, the risk of *H. pylori* infection rises significantly (*p* < 0.05). Conversely, there was no linear relationship between T4 levels and *H. pylori* infections.

## 4 Discussion

This study utilized newly updated NHANES data to explore the relationship between TSH and T4 levels and *H. pylori* infection. In summary, *H. pylori* seropositivity demonstrated a positive correlation with T4 levels and a negative correlation with TSH levels. In stratified analyses, the adjusted association between serum T4 levels and *H. pylori* seropositivity was statistically significant in men but not in women, with significant correlations observed for serum TSH in participants over 68 years of age and for T4 in those aged 41–45 years.

As is widely acknowledged, thyroid function is composed of TSH and T4, with these serum markers often exhibiting a negative correlation. Our research identified variations in TSH and T4 levels between populations positive and negative for *H. pylori*. The non-linear connection revealed an enormous rise in the likelihood of *H. pylori* infection when TSH levels < 0.98 IU/mL. This suggests that individuals exhibiting hyperthyroid tendencies are more susceptible to *H. pylori* infection, corroborating findings from prior studies (10).

*H. pylori* testing should be considered for individuals undergoing treatment with medications known to be affected by this infection, such as T4, as outlined in the Houston Consensus (23). Therefore, this suggests an implied relationship between *H. pylori* infection and thyroid function. Nonetheless, the impact of thyroid function disorders, including hyperthyroidism or hypothyroidism, on *H. pylori* infection remains a contentious issue. Larizza et al. demonstrated that *H. pylori* could provoke an immune response against thyroid cells (24). A notable association has also been observed between Cag-A-positive *H. pylori* strains and GD, regardless of the patients’ hormonal status (25). The association may be attributed to cross-reactivity between antibodies against the *H. pylori* Cag-A protein and the follicular cells of the thyroid gland (26). Additionally, conflicting findings from other research have indicated a positive correlation between *H. pylori* infection and autoimmune atrophic thyroiditis (27, 28).

The mechanisms by which elevated T4 levels and suppressed TSH levels heighten the susceptibility to *H. pylori* infection remain incompletely understood; however, several lines of evidence may shed light on this relationship. Firstly, the discovery of a homologous 11-residue peptide shared by both gastric parietal cell antigens and thyroid peroxidase suggests a common epitope (29), implying that antibodies generated during *H. pylori* infection may cross-react with thyroid antigens, potentially contributing to hyperthyroidism (30). Secondly, studies have shown a strong correlation between IgG anti-*H. pylori* antibodies and thyroid autoantibodies, along with a reduction in thyroid autoantibody levels following the successful eradication of *H. pylori* infection (31). Thirdly, previous investigations have demonstrated that *H. pylori* strains can express fucosylated Lewis determinants, which are commonly found in various host tissues and may trigger an autoimmune response that could impair thyroid function (32). Typically, thyroid diseases, particularly ATDs, are influenced by a variety of autoimmune mechanisms. It has been reported that *H. pylori* infection plays a role in the pathogenesis of HT, the leading cause of hypothyroidism (28). In addition, in GD, humoral autoimmunity characterized by a TH2 profile is prevalent. Once infected with *H. pylori*, cytokines such as interleukin (IL)-4, IL-5, and IL-6 are activated, initiating a cascade of humoral immune responses that may modify the expression of adhesion molecules on the gastric mucosa, thereby contributing to the hyperthyroidism observed in GD (10, 33). The mechanism is believed to be associated with molecular mimicry between *H. pylori* antigens and thyroid constituents. Collectively, these findings offer compelling explanations for the observed correlation between *H. pylori* infection and hyperthyroidism.

A notable correlation was found between persistent *H. pylori* infection and ATDs in female patients, regardless of thyroid gland functional status. Specifically, this association was observed in relation to both HT and GD across multiple studies (10, 12), whereas no such connection was noted for non-autoimmune thyroid disorders. Although this association was primarily observed in women, a trend suggesting a similar relationship was noted in men (34). Because of the limitations of the NHANES database, the present study was unable to include thyroid autoantibodies such as thyroglobulin antibody (TGAb) and thyroid peroxidase antibodies (TPOAbs), which may have resulted in a weakened association between *H. pylori* seropositivity and TSH levels as well as T4 levels within gender cohorts.

Previous studies have suggested that the successful colonization of *H. pylori* in the gastric environment is influenced by age-related physiological factors and host characteristics, with the incidence of *H. pylori*-related diseases increasing with age (35, 36), a finding consistent with our results. Furthermore, additional factors such as socioeconomic status, geographical location, and ethnicity may also contribute to the rates of *H. pylori* infection (37). Breckan et al. reported that the prevalence of *H. pylori* is age-dependent, rising from adolescence and reaching its peak between the ages of 60 and 70 years (38).

It has to be recognized that there are several limitations in this study. First, *H. pylori* infection was defined based on serological testing, which cannot distinguish between past and present infections. Second, the NHANES 1999–2000 dataset lacked certain relevant information, such as TGAb and TPOAb, which weakens the association between *H. pylori* and thyroid autoimmunity, thus hindering a more thorough exploration of the underlying mechanisms. Should future NHANES updates include these data, more detailed classification comparisons could be conducted again in the future. Third, as a cross-sectional study, it does not allow for the establishment of causative relationships between TSH and T4 levels and *H. pylori* seropositivity, nor does it provide temporal data to differentiate between past and present *H. pylori* infections. Further longitudinal studies are needed to make interpretations of observed associations in the future. Lastly, while we accounted for the influence of certain medications, other factors, such as long-term contraceptive use in women, may affect thyroid function (39), as well as dietary factors like “high-salt” intake (40) or a high dietary inflammatory index (41), which are likely to influence the prevalence of *H. pylori* infection.

## 5 Conclusion

Overall, serum TSH and T4 levels were found to be associated with the risk of *H. pylori* infection, particularly among men, individuals with hyperthyroidism, and elderly adults. These people should pay close attention to *H. pylori* screening, as *H. pylori* infection is closely related to gastric cancer.

## Data Availability

The raw data supporting the conclusions of this article will be made available by the authors, without undue reservation.

## References

[1] Zamani M Ebrahimtabar F Miller V Miller W.H Alizadeh-Navael R Shokri-Shirvanl J. Systematic review with meta analysis: the worldwide prevalence of Helicobacter pylori infection. Aliment Pharmacol Ther. (2018) 47:868–76. doi: 10.1111/apt.2018.47.issue-7 29430669

[2] Burucoa C Axon A. Epidemiology of Helicobacter pylori infection. Helicobacter. (2017) 22:1–5. doi: 10.1111/hel.2017.22.issue-S1 28891138

[3] Astl J Sterzl I. Activation of helicobacter pylori causes either autoimmune thyroid diseases or carcinogenesis in the digestive tract. Physiol Res. (2015) 64:S291–301. doi: 10.33549/physiolres 26680492

[4] Kucukazman M Yeniova O Dal K Yavuz B. Helicobacter pylori and cardiovascular disease. Eur Rev Med Pharmacol Sci. (2015) 19:3731–41. 26502864

[5] Wang L Cao Z-M Zhang L-L Dai X-C Liu Z-J Zeng Y-X. Helicobacter pylori and autoimmune diseases: Involing multiple systems. Front Immunol. (2022) 13:833424. doi: 10.3389/fimmu.2022.833424 35222423 PMC8866759

[6] Mullur R Liu YY Brent GA. Thyroid hormone regulation of metabolism. Physiol Rev. (2014) 94:355–82. doi: 10.1152/physrev.00030.2013 PMC4044302 24692351

[7] Chiamolera MI Wondisford FE. Minireview: thyrotropin-releasing hormone and the thyroid hormone feedback mechanism. Endocrinology. (2009) 150:1091–6. doi: 10.1210/en.2008-1795 19179434

[8] Wang Y Zhu S Xu Y Wang X Zhu Y. Interaction between gene a-positive helicobacter pylori and human leukocyte antigen II alleles increase the risk of graves disease in Chinese han population: An association study. Gene. (2013) 531:84–9. doi: 10.1016/j.gene.2013.07.069 23954255

[9] Hou Y Sun W Zhang C Wang T Guo X Wu L. Meta-analysis of the correlation between helicobacter pylori infection and autoimmune thyroid diseases. Oncotarget. (2017) 8:115691–700. doi: 10.18632/oncotarget.22929 PMC5777804 29383192

[10] Bassi V Marino G Iengo A Fattoruso O Santinelli C. Autoimmune thyroid diseases and Helicobacter pylori: The correlation is present only in Graves’s disease. World J Gastroenterol. (2012) 18:1093–7. doi: 10.3748/wjg.v18.i10.1093 PMC3296983 22416184

[11] Shi WJ Liu W Zhou XY Ye F Zhang GX. Associations of helicobacter pylori infection and cytotoxin-associated gene a status with autoimmune thyroid diseases: a meta-analysis. Thyroid. (2013) 23:1294–300. doi: 10.1089/thy.2012.0630 23544831

[12] Zhang J Hai X Wang S Zhu F Gu Y Meng G. Helicobacter pylori infection increase the risk of subclinical hyperthyroidism in middle-aged and elderly women independent of dietary factors: Results from the Tianjin chronic low-grade systemic inflammation and health cohort study in China. Front Nutr. (2023) 10:1002359. doi: 10.3389/fnut.2023.1002359 36950328 PMC10025335

[13] Fan H Liu Z Zhang X Wu S Shi T Zhang P. Thyroid stimulating hormone levels are associated with genetically predicted nonalcoholic fatty liver disease. J Clin Endocrinol Metab. (2022) 107:2522–9. doi: 10.1210/clinem/dgac393 35763044

[14] Skelin M Lucijanić T Amidžić Klarić D Resič A Bakula M LiberatiČizmek AM. Factors affecting gastrointestinal absorption of levothyroxine: A review. Clin Ther. (2017) 39:378–403. doi: 10.1016/j.clinthera.2017.01.005 28153426

[15] Triantafifillidis JK Georgakopoulos D Gikas A Merikas E Peros G Sofroniadou K. Relation between helicobacter pylori infection, thyroid hormone levels and cardiovascular risk factors on blood donors. Hepatogastroenterology. (2003) 50:cccxviii–cccxx. 15244214

[16] Tomasi PA Dore MP Fanciulli G Sanciu F Realdi G Delitala G. Is there anything to the reported association between helicobacter pylori infection and autoimmune thyroiditis? Dig Dis Sci. (2005) 50:385–8. doi: 10.1007/s10620-005-1615-z 15745105

[17] Prevention CfDCa. National health and nutrition examination survey: centers for disease control and prevention (2020). Available online at: https://www.cdc.gov/nchs/nhanes/index.htm (Accessed 05 Apr 2021).

[18] Huang J Liu Z Ma J Liu J Lv M Wang F. The association between helicobacter pylori seropositivity and bone mineral density in adults. Med Inflammation. (2022) 2022:2364666. doi: 10.1155/2022/2364666 PMC9001096 35418807

[19] Centers for Disease Control and Prevention. National Health and Nutrition Examination Survey 1999–2001 Data Documentation, Codebook, and Frequencies – H. pylori . Available online at: https://wwwn.cdc.gov/Nchs/Nhanes/1999-2000/LAB11.htm (Accessed 1 April 2024).

[20] Berrett AN Gale SD Erickson LD Brown BL Hedges DW. Folate and inflammatory markers moderate the association between helicobacter pylori exposure and cognitive function in US adults. Helicobacter. (2016) 21:471–80. doi: 10.1111/hel.12303 26935014

[21] Meier HCS Miller FW Dinse GE Weinberg CR Cho CC Parks CG. Helicobacter pylori seropositivity is associated with antinuclear antibodies in US adults, NHANES 1999-2000. Epidemiol Infect. (2020) 148:e20. doi: 10.1017/S0950268820000126 32019616 PMC7019483

[22] Huang JW Xie C Niu Z He LJ Li JJ. The relation between helicobacter pylori immunoglobulin G seropositivity and leukocyte telomere length in US adults from NHANES 1999-2000. Helicobacter. (2020) 25:e12760. doi: 10.1111/hel.12760 33002310

[23] El-Serag HB Kao JY Kanwal F Gilger M LoVecchio F Moss SF. Houston Consensus conference on testing for helicobacter pylori infection in the United States. Clin Gastroenterol Hepatol. (2018) 16:992–1002. doi: 10.1016/j.cgh.2018.03.013 29559361 PMC6913173

[24] Larizza D Calcaterra V Martinetti M Negrini R De Silvestri A Cisternino M. Helicobacter pylori infection and autoimmune thyroid disease in young patients: the disadvantage of carrying the human leukocyte antigenDRB1*0301 allele. J Clin Endocrinol Metab. (2006) 91:176–9. doi: 10.1210/jc.2005-1272 16263823

[25] Bassi V Santinelli C Iengo A Romano C. Identification of a correlation between Helicobacter pylori infection and Graves’ disease. Helicobacter. (2010) 15:558–62. doi: 10.1111/j.1523-5378.2010.00802.x 21073613

[26] Figura N Di Cairano G Moretti E Lacoponi F Santucci A Bernardini G. Helicobacter pylori infection and autoimmune thyroid diseases: the role of virulent strains. Antibiotics. (2019) 9:12. doi: 10.3390/antibiotics9010012 31906000 PMC7167994

[27] Papamichael KX Papaioannou G Karga H Roussos A Mantzaris GJ. Helicobacter pylori infection and endocrine disorders: is there a link? World J Gastroenterol. (2009) 15:2701–7. doi: 10.3748/wjg.15.2701 PMC2695884 19522019

[28] De Luis DA Varela C de la Calle H Cantón R de Argila CM San Roman AL. Helicobacter pylori infection is markedly increased in patients with autoimmune atrophic thyroiditis. J Clin Gastroenterol. (1998) 26:259–63. doi: 10.1097/00004836-199806000-00008 9649006

[29] Elisei R Mariotti S Swillens S Vassart G Ludgate M. Studies with recombinant autoepitopes of thyroid peroxidase: evidence suggesting an epitope shared between the thyroid and the gastric parietal cell. Autoimmunity. (1990) 8:65–70. doi: 10.3109/08916939008998434 1717010

[30] Choi YM Kim TY Kim EY Jang EK Jeon MJ Kim WG. Association between thyroid autoimmunity and helicobacter pylori infection. Korean J Intern Med. (2017) 32:309–13. doi: 10.3904/kjim.2014.369 PMC5339455 28092700

[31] Bertalot G Montresor G Tampieri M Spasiano A Pedroni M Milanesi B. Decrease in thyroid autoantibodies after eradication of helicobacter pylori infection. Clin Endocrinol. (2004) 61:650–2. doi: 10.1111/j.1365-2265.2004.02137.x 15521972

[32] Wirth HP Yang M Karita M Blaser MJ. Expression of the human cell surface glycoconjugates Lewis x and Lewis y by helicobacter pylori isolates is related to cagA status. Infect Immun. (1996) 64:4598–605. doi: 10.1128/iai.64.11.4598-4605.1996 PMC174419 8890213

[33] Roura-Mir C Catálfamo M Sospedra M Alcalde L PujolBorrell R Jaraquemada D. Single-cell analysis of intrathyroidal lymphocytes shows differential cytokine expression in Hashimoto’s and Graves’ disease. Eur J Immunol. (1997) 27:3290–302. doi: 10.1002/eji.1830271228 9464817

[34] Dore MP Fanciulli G Manca A Pes GM. Association of helicobacter pylori infection with autoimmune thyroid disease in the female sex. J Clin Med. (2023) 12:5150. doi: 10.3390/jcm12155150 37568552 PMC10419966

[35] Huang JY Sweeney EG Sigal M Zhang HC Remington SJ Cantrell MA. Chemodetection and destruction of host urea allows helicobacter pylori to locate the epithelium. Cell Host Microbe. (2015) 18:147–56. doi: 10.1016/j.chom.2015.07.002 PMC4593702 26269952

[36] Goh KL Chan WK Shiota S Yamaoka Y. Epidemiology of helicobacter pylori infection and public health implications. Helicobacter. (2011) 16:1–9. doi: 10.1111/j.1523-5378.2011.00874.x PMC3719046 21896079

[37] Sreeja SR Le T-D Eom BW Oh S-H Shivappa N Hebert J-R. Association between the dietary inflammatory index and gastric disease risk: findings from a Korean population-based cohort study. Nutrients. (2022) 14:2662. doi: 10.3390/nu14132662 35807849 PMC9268659

[38] Breckan RK Paulssen EJ Asfeldt AM Kvamme J-M Straume B Florholmen J. The all-age prevalence of helicobacter pylori infection and potential transmission routes. A population-based study. Helicobacter. (2016) 21)6:586–95. doi: 10.1111/hel.12316 27172105

[39] Qiu Y Hu Y Xing Z Fu Q Zhu J Su A. Birth control pills and risk of hypothyroidism: a cross-sectional study of the national health and nutrition examination survey, 2007-2012. BMJ Open. (2021) 11:e46607. doi: 10.1136/bmjopen-2020-046607 PMC8230965 34162647

[40] Shu L Zheng P-F Zhang X-Y Feng Y-L. Dietary patterns and Helicobacter pylori infection in a group of Chinese adults ages between 45 and 59 years old. Medicine. (2019) 98:2(e14113). doi: 10.1097/MD.0000000000014113 PMC6336658 30633225

[41] Xiong YJ Du LL Diao YL Wen J Meng XB Gao J. Association of dietary inflammatory index with helicobacter pylori infection and mortality among US population. J Trans Med. (2023) 21:538. doi: 10.1186/s12967-023-04398-8 PMC10422799 37573314

